# Racial Differences in Arterial Stiffness and Microcirculatory Function Between Black and White Americans

**DOI:** 10.1161/JAHA.112.002154

**Published:** 2013-04-24

**Authors:** Alanna A. Morris, Riyaz S. Patel, Jose Nilo G. Binongo, Joseph Poole, Ibhar al Mheid, Yusuf Ahmed, Neli Stoyanova, Viola Vaccarino, Rebecca Din‐Dzietham, Gary H. Gibbons, Arshed Quyyumi

**Affiliations:** 1 Division of Cardiology, Emory University School of Medicine, Atlanta, GA (A.A.M., R.S.P., J.P., I.M., Y.A., V.V., A.Q.); 2 Cardiovascular Research Institute, Morehouse School of Medicine, Atlanta, GA (N.S., R.D.D.); 3 Division of Epidemiology, Emory Rollins School of Public Health, Atlanta, GA (V.V.); 4 Department of Biostatistics and Bioinformatics, Emory Rollins School of Public Health, Atlanta, GA (J.N.G.B.); 5 National Heart, Lung and Blood Institute, National Institutes of Health, Bethesda, MD (G.H.G.)

**Keywords:** arterial stiffness, racial disparities, reactive hyperemia

## Abstract

**Background:**

Compared with whites, black Americans suffer from a disproportionate burden of cardiovascular disease (CVD). We hypothesized that racial differences in the prevalence of CVD could be attributed, in part, to impaired vascular function in blacks after adjustment for differences in risk factor burden.

**Methods and Results:**

We assessed vascular function in 385 black and 470 white subjects (mean age, 48±11 years; 45% male). Using digital pulse amplitude tonometry (EndoPAT) we estimated the reactive hyperemia index (RHI), a measure of microvascular endothelial function, and peripheral augmentation index (PAT‐AIx). Central augmentation index (C‐AIx) and pulse‐wave velocity (PWV) were measured as indices of wave reflections and arterial stiffness, respectively, using applanation tonometry (Sphygmocor). Compared with whites, blacks had lower RHI (2.1±0.6 versus 2.3±0.6, *P*<0.001), greater arterial wave reflections assessed as both PAT‐AIx (20.4±21.5 versus 17.0±22.4, *P*=0.01) and CAIx (20.8±12.3 versus 17.5±13.3, *P*=0.001), and greater arterial stiffness, measured as PWV (7.4±1.6 versus 7.1±1.6 m/s, *P*=0.001). After adjustment for traditional CVD risk factors, black race remained a significant predictor of lower RHI and higher PAT‐AIx and CAIx (all *P*<0.001) in all subjects and of higher PWV in men (*P*=0.01). Furthermore, these associations persisted in a subgroup analysis of “healthy” individuals free of CVD risk factors.

**Conclusion:**

Black race is associated with impaired microvascular vasodilatory function, and greater large arterial wave reflections and stiffness. Because impairment in these vascular indices may be associated with worse long‐term outcomes, they may represent underlying mechanisms for the increased CVD risk in blacks.

## Introduction

Despite an overall trend toward decreasing mortality from cardiovascular disease (CVD) over recent decades in the United States, blacks continue to experience disproportionately higher CVD morbidity and mortality when compared with whites.^1^ These observations are only partly explained by a higher prevalence of traditional CVD risk factors such as obesity, hypertension, type 2 diabetes mellitus, and tobacco use among blacks.^2–3^ Assessments of subclinical disease in microcirculation and in large arteries by vascular function testing may provide valuable insights into the etiology of this health disparity.

In the healthy state, the endothelium maintains vascular tone and homeostasis through mediators such as nitric oxide (NO).^4–5^ Endothelial dysfunction, characterized as decreased NO bioavailability from exposure to CVD risk factors, ultimately leads to the development and progression of atherosclerosis with its sequelae of myocardial infarction and stroke.^6–7^ Endothelial functional assessments by traditional approaches using intra‐arterial agonists or flow‐mediated vasodilation are impractical for deployment in population studies. Emerging data support the use of digital pulse amplitude tonometry (PAT) for the noninvasive assessment of microvascular function that can be assessed rapidly and reliably by measuring pulse amplitude in the fingertip at rest and following the induction of reactive hyperemia.^5^ Lower PAT hyperemic response correlates with the presence of CVD risk factors^8–9^ and with brachial and coronary arterial endothelial dysfunction.^9–11^ Importantly, approximately half of the PAT hyperemic response is mediated by NO.^12^

Arterial stiffening is also associated with CVD risk factors and may worsen hypertension through a feed‐forward mechanism, with chronic elevations in blood pressure leading to arterial wall thickening and compensatory remodeling, adversely affecting the internal elastic properties of the vessel wall.^13^ Noninvasive and reproducible techniques allow estimation of central aortic pressures and stiffness.^14–15^ Aortic pulse‐wave velocity (PWV), an estimate of the speed of the pressure wave traveling along the aorta, is regarded as a direct measure of large artery stiffness. The augmentation index (AIx), a composite measure of the magnitude of arterial wave reflections and systemic arterial stiffness, increases as PWV increases. Impaired arterial elastic properties, measured as the aortic AIx and/or PWV, are increasingly recognized as independent predictors of incident CVD events (myocardial infarction, stroke, revascularization), as well as all‐cause mortality.^16^

Prior studies examining the relationship between race and vascular function have often been restricted to relatively small cohorts using invasive and/or cumbersome techniques to assess arterial health. Blacks were found to have impaired endothelium‐dependent and ‐independent vasodilation compared with whites.^17–18^ Moreover, endothelial cells of blacks appear to generate more oxidant stress, leading to enhanced nitric oxide (NO) inactivation.^19–20^ This diminished response to endogenous and exogenous NO in blacks may partly account for the more severe hypertension in this population. In addition, blacks appeared to have greater arterial stiffness, although many of these analyses did not fully account for differences in risk factor burden.^21–22^ Impairment of vascular function may represent a mechanism through which increased cardiovascular risk is manifested. We hypothesized that, compared with whites, blacks would have impaired microvascular function, increased arterial stiffness, and abnormal wave reflections that would be independent of differences in CVD risk factor burden.

## Methods

### Study Sample

Self‐identified black and white residents of metropolitan Atlanta, aged 20 to 70 years (n=929) without a history of preexisting CVD, were recruited from March 2005 to October 2009 to come to either the Emory or Morehouse Schools of Medicine for evaluation. Detailed information on demographics and anthropometrics was collected. Blood pressure was measured with a sphygmomanometer after 5 minutes of rest and was based on the average of the final 2 of 3 readings measured 5 minutes apart. Height and weight were measured, and body mass index (BMI) was calculated as weight in kilograms divided by height in meters squared (kg/m^2^). History of diabetes and hypertension was defined by participant self‐report, or use of antidiabetic or antihypertensive medications. Smoking history, obtained using standardized questionnaires, was defined as current or never/former (no cigarettes within the past 30 days). Pregnant women, participants with history of myocardial infarction or stroke, and those with acute illnesses were excluded. The study was approved by the Emory University and Morehouse School of Medicine Institutional Review Committees. Informed consent was obtained from all participants.

### Blood Specimens

Participants were instructed to fast and to refrain from smoking for 12 hours before the study visit. Venous blood was collected in sodium heparin tubes. Serum levels of total cholesterol, low‐density lipoprotein cholesterol (LDL‐C), high‐density lipoprotein cholesterol (HDL‐C), triglycerides, and glucose were measured by spectrophotometry.

### Pulse Volume Analysis

**Figure 1.**
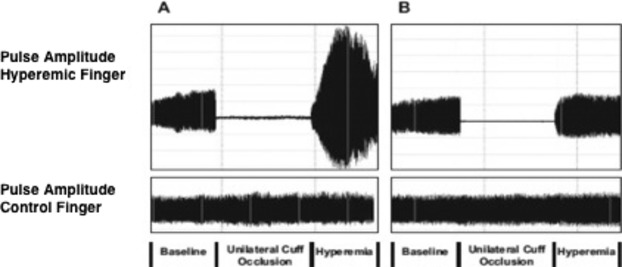
Digital pulse amplitude in a participant with a pulse amplitude tonometry (PAT) ratio in the highest tertile (A) and in a different participant with a PAT ratio in the lowest tertile (B). In the arm undergoing hyperemia (top tracing, A and B), baseline pulse amplitude is recorded. Flow is occluded in both participants during cuff inflation; subsequently, flow rises rapidly after cuff release in the participant with a high response (A) but not in the participant with a low response (B) during the hyperemic period. In the contralateral control finger (bottom tracing, A and B), flow continues throughout, and pulse amplitude undergoes minimal change. Adapted with permission from Hamburg et al.^8^

Digital pulse amplitude tonometry (PAT) was used to measure pulse volume amplitude (PVA) in the tip of the index finger, with participants resting in the supine position in a quiet, temperature‐controlled environment set at 22°C after an overnight fast (Endo‐PAT, Itamar‐Medical, Israel). Full details of the probe technology and the basis of measurements are available elsewhere.^10,23^ PVA was analyzed at rest and during reactive hyperemia, which was elicited by the release of an upper arm blood pressure cuff inflated to suprasystolic pressure for 5 minutes. The reactive hyperemia index (RHI) was calculated as the ratio of the post‐ to preocclusion PVA of the tested arm, divided by the post‐ to preocclusion ratio of the control arm (the average PVA over a 1‐minute interval starting 1‐minute after cuff deflation divided by the average PVA measured for 1 minute before cuff inflation [baseline])^10–11,23^ (Figure 1). The Framingham RHI (fRHI) was also calculated on the basis of previously described methods.^8^ An augmentation index (PAT‐AIx), representing an estimate of peripheral wave reflections, was also derived from the 2 systolic peaks of the baseline PAT waveform. Participants were excluded from this analysis for missing values (n=68) or technically inadequate study (n=6), leaving n=855 in the final analysis. Compared with those who were included in the analysis, among the 74 participants excluded from the analysis there was a higher percentage of women (67% versus 55%, *P*<0.001) and African Americans (59% versus 45%, *P*<0.001).

### Arterial Waveform Analysis

Indices of arterial stiffness and wave reflections were estimated in the supine position after an overnight fast using the Sphygmocor device (Atcor Medical, Australia), which records sequential high‐quality pressure waveforms at peripheral pulse sites using a high‐fidelity tonometer. Full details of the probe technology and basis of measurements are available elsewhere.^24^

Pulse‐wave analysis was performed on the basis of acquisition of radial artery pressure waveforms with application of a generalized transfer function to derive the central aortic pressure waveform, from which estimates of central pulse pressure (CPP) are generated. The central augmentation index (CAIx) is a function of the degree of pressure augmentation (AP) secondary to reflected waves from the periphery (AIx=AP/total CPP). CAIx standardized to a heart rate of 75 bpm was calculated and used for the purposes of this study.^25^

Pulse‐wave velocity (PWV) measured between carotid and femoral arteries is a regional assessment of aortic stiffness and is the gold standard index of arterial stiffness.^24^ Pressure waveforms at the carotid and femoral arterial sites were acquired using tonometry and electrocardiographic gating. Velocity (distance/time in meters/second) was calculated using the “foot‐to‐foot” method, measuring the interval between the R wave on the ECG and the foot of the recorded pressure waveform at each site, whereas distance between the sites was measured manually by the operator. Data on PWV were available for 577 participants. Compared with the total population, there was a higher percentage of blacks (56% versus 40%, *P*<0.001) and women (64% versus 52%, *P*=0.001) in the 278 participants who were missing data on PWV. Reproducibility studies in our laboratory on consecutive days have demonstrated a coefficient of variation of 20.3% and 3.8% for CAIx and PWV, respectively.

### Statistical Methods

Study variables are described as the mean±standard deviation (SD) for normally distributed continuous variables, median (interquartile range) for skewed continuous variables, or proportions for categorical variables. RHI, fRHI, PAT‐AIx, CAIx, and PWV were examined as continuous variables. All continuous variables were first tested for normality using the Kolmogorov–Smirnov criterion. Groups were compared using the chi‐square test for categorical outcomes and *t* tests or Wilcoxon rank sum tests for continuous outcomes.

Multivariable linear regression models were constructed to examine the association of race with indices of vascular function after adjusting for risk factors. Risk factors were selected on the basis of their known association with vascular function and included age, sex, socioeconomic status (level of education), history of hypertension, history of diabetes, smoking status, BMI, mean arterial pressure, triglycerides, LDL‐C, ratio of total/HDL cholesterol, and glucose. Predefined subgroup analyses were performed in participants who were free of traditional risk factors for CVD. All tests of statistical significance were 2‐tailed, and *P*<0.05 was considered significant. Statistical analyses were performed using SPSS, Inc, v19.0 (Chicago, IL).

## Results

### Subject Characteristics

Demographic and clinical characteristics of the study participants are presented in Table 1. The mean age was 48±11 years, 55% were female, and 45% were black. Compared with whites, blacks were younger, were more likely to have a history of hypertension, diabetes, and smoking, and were less likely to be college graduates. Blacks had higher BMI and mean arterial pressure and lower levels of triglycerides than whites.

**Table 1.** Subject Characteristics by Racial Group

	Total Population (n=855)	Whites (n=469)	Blacks (n=386)	*P* Value
Age, y	48±11	49±11	47±10	0.005
Female, %	474 (55)	275 (59)	199 (52)	0.83
History of hypertension, %	218 (28)	91 (21)	127 (37)	<0.001
History of diabetes, %	54 (7)	16 (4)	38 (11)	<0.001
Current smoking, %	118 (14)	35 (8)	83 (23)	<0.001
Education*				<0.001
High school or GED	135 (21)	35 (10)	100 (31)	
Some college	164 (25)	62 (19)	102 (32)	
College graduate	354 (54)	235 (71)	119 (37)	
Body mass index, kg/m^2^	27 (23, 32)	25 (23, 29)	29 (25, 35)	<0.001
Mean arterial pressure, mm Hg	89 (82, 97)	88 (81, 96)	92 (84, 100)	<0.001
Total/HDL‐C	3 (3, 4)	3 (3, 4)	3 (3, 4)	0.73
Triglycerides, mg/dL	90 (65, 125)	95 (66, 133)	86 (63, 113)	<0.001
LDL‐C, mg/dL	113±33	112±31	115±36	0.55
Glucose, mg/dL	87 (82, 93)	88 (82, 94)	87 (81, 93)	0.30
Framingham risk, %				0.9
Low	719 (86)	401 (86)	318 (86)	
Intermediate	100 (12)	56 (12)	44 (12)	
High	14 (2)	7 (2)	7 (2)	

Values shown are mean±SD, median (interquartile range), or n (%). GED indicates General Educational Development; HDL‐C, high‐density lipoprotein cholesterol; LDL‐C, low‐density lipoprotein cholesterol; SD, standard deviation. *P* values are for black–white comparison.

* Data on level of education available for n=653 participants.

### Clinical Correlates of PAT and Arterial Stiffness

Table 2 depicts the associations of traditional CVD risk factors with indices of vascular function, after adjusting for race, sex, age, smoking, history of hypertension or diabetes, BMI, mean arterial pressure, lipids, and glucose. RHI positively correlated with age and LDL‐C, but was negatively correlated with total/HDL cholesterol. fRHI was negatively correlated with smoking, BMI, and total/HDL cholesterol. PAT‐AIx and CAIx positively correlated with female sex, age, mean arterial pressure, but were negatively correlated with total/HDL cholesterol. PAT‐AIx also positively correlated with smoking and trended toward correlation with history of hypertension (*P*=0.06), but was negatively correlated with history of diabetes and BMI. CAIx also positively correlated with history of hypertension, triglycerides, and LDL‐C. PWV positively correlated with age, history of diabetes, and mean arterial pressure, but was negatively correlated with LDL‐C.

**Table 2.** Multivariate Linear Regression of CVD Risk Factors With RHI and Indices of Arterial Elasticity

	RHI	fRHI	PAT‐AIx	CAIx	PWV
Black race	−0.170**	−0.020	0.127**	0.170**	0.082
Female sex	−0.028	0.046	0.117**	0.162**	0.013
Age	0.095**	0.027	0.475**	0.462**	0.264**
Current smoking	−0.013	−0.082**	0.110**	0.057	−0.047
History of hypertension	0.015	0.025	0.068	0.073**	0.041
History of diabetes	0.007	0.018	−0.083**	−0.029	0.111**
Body mass index, kg/m^2^	−0.025	−0.101**	−0.144**	−0.051	−0.089
Mean arterial pressure, mm Hg	−0.031	−0.029	0.127**	0.190**	0.312**
Total/HDL‐C	−0.146**	−0.238**	−0.197**	−0.239**	0.086
Triglycerides, mg/dL	0.031	−0.003	0.066	0.180**	−0.011
LDL‐C, mg/dL	0.149**	0.199	0.067	0.099**	−0.128**
Glucose, mg/dL	−0.027	−0.069	0.008	−0.045	0.018

Values shown are standardized beta coefficients. Values are adjusted for all CVD risk factors displayed in the first column. CVD indicates cardiovascular disease; RHI, reactive hyperemia index; fRHI, Framingham reactive hyperemia index; PAT‐AIx, peripheral augmentation index; CAIx, central augmentation index; PWV, pulse‐wave velocity; HDL‐C, high‐density lipoprotein cholesterol; LDL‐C, low‐density lipoprotein cholesterol.

* *P*≤0.05

** *P*≤0.001.

### Race‐Related Differences in Microvascular Function

**Figure 2.**
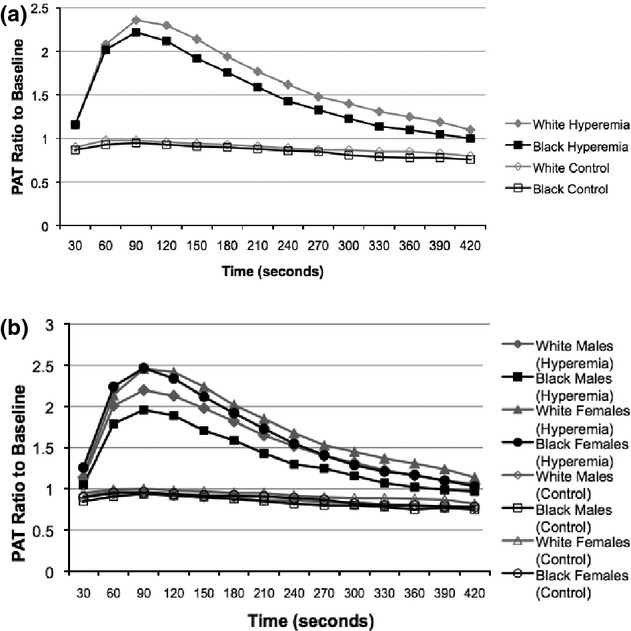
A, Pulse amplitude response shown for the hyperemic finger and control finger in whites and blacks. Peak hyperemia occurred at 90 seconds in both groups. Blacks had lower responses from peak hyperemia throughout in both fingers (*P*≤0.01). Values are means. The minimum and maximum SEs were 0.02 to 0.06. B, Pulse amplitude response shown for the hyperemic finger and control finger in whites and blacks stratified by sex. Blacks had lower responses from peak hyperemia throughout in both fingers. Values are means. The minimum and maximum SEs were 0.02 to 0.09. PAT indicates pulse amplitude tonometry; SE, standard error of the mean.

RHI was significantly lower in blacks than whites (Table 3). Furthermore, blacks were more likely to have RHI <1.67 (21.4% versus 13.5%, *P*=0.003), a value that has previously been associated with coronary endothelial dysfunction.^11^ Baseline digital pulse volume amplitude (PVA) was also significantly lower in blacks compared with whites (Table 3). Although the peak fingertip hyperemic response was similar in blacks and whites, hyperemia during recovery from the peak response remained significantly lower in blacks at all times (*P*≤0.01; Figure 2A). After stratifying by sex, the findings were similar; white women had the highest hyperemic response, whereas black men had the lowest (Figure 2B). After multivariable adjustment for age, sex, history of hypertension, history of diabetes, smoking status, BMI, mean arterial pressure, triglycerides, LDL‐C, ratio of total/HDL cholesterol, and glucose, black race remained an independent predictor of lower RHI (β=−0.169, *P*<0.001) and lower baseline PVA (β=−0.143, *P*<0.001).

**Table 3.** Measures of Vascular Function by Racial Group Adjusted for CVD Risk Factors

	Baseline PVA^†^	RHI	fRHI	PAT‐AIx	CAIx	PWV
Blacks	275±6**	2.1±0.04**	0.8±0.01	21.4±1.1**	21.2±0.6**	7.3±0.1**
Whites	328±6	2.3±0.03	0.8±0.01	15.7±1.0	16.6±0.6	7.1±0.1

Values are mean±standard error. Values are adjusted for race, sex, age, smoking, history of hypertension or diabetes, BMI, mean arterial pressure, lipids, and glucose. CVD indicates cardiovascular disease; PVA, pulse volume amplitude; RHI, reactive hyperemia index; fRHI, Framingham reactive hyperemia index; PAT‐AIx, peripheral augmentation index; CAIx, central augmentation index; PWV, pulse‐wave velocity; BMI, body mass index.

*P≤0.01, **P≤0.001 when compared with whites.

^†^Values shown are in occluded arm.

### Race‐Related Differences in Wave Reflections and Arterial Stiffness

As shown in Table 3, blacks had higher PAT‐AIx, CAIx, and PWV compared with whites. After adjusting for risk factors, black race remained an independent predictor of higher PAT‐AIx (β=0.127, *P*<0.001) and CAIx (β=0.170, *P*<0.001). In multivariable models stratified by sex, black race remained an independent predictor of higher PWV in men (β=0.153, *P*=0.02) but not women (β=0.037, *P*=0.60), although the formal test for race×sex interaction was not significant (*P*=0.40).

### Subgroup Analysis in Low‐Risk Participants

For this analysis, participants were excluded for history of hypertension or diabetes, current smoking, measured SBP ≥140 or DBP ≥90 mm Hg, BMI ≥30 kg/m^2^, or fasting glucose ≥100 mg/dL. In the 390 participants who were free of these traditional risk factors for CVD, blacks (n=123) had lower RHI (2.1±0.6 versus 2.4±0.6, *P*<0.001), and higher PWV (7.3±1.6 versus 6.8±1.4, *P*=0.002). There was no univariate difference in PAT‐AIx or CAIx. After adjusting for risk factors including age, sex, and lipids, black race remained an independent predictor of lower RHI (β=−0.149, *P*=0.006) and higher PWV (β=0.188, *P*=0.001) and CAIx (β=0.136, *P*=0.003).

## Discussion

In this biracial community‐based sample, we demonstrate that black race is associated with impaired vascular function compared with whites. Specifically, blacks had (1) reduced microvascular endothelial function measured as RHI; (2) abnormal arterial wave reflections, measured as higher PAT‐AIx and CAIx; and (3) greater arterial stiffness, measured as higher PWV, even after adjustment for traditional CVD risk factors. Importantly, these differences were present even in the subgroup of participants who were completely free of conventional CVD risk factors.

Mechanisms underlying the observed ethnic differences in vascular function may involve both NO‐dependent and ‐independent mechanisms. NO is tonically released from endothelial cells and is essential to the maintenance of vasodilator tone and homeostasis, which are adversely affected by CVD risk factors.^4,7,26^ Decreased NO bioavailability can lead to vascular remodeling in experimental models, affecting both large elastic arteries and smaller resistance vessels.^27–28^ We and others have shown that NO contributes to the total duration of reactive hyperemia but not to peak hyperemia, and that the RHI is in part NO‐dependent.^12,29^ In addition, using invasive techniques, we and others have shown that blacks have reduced NO bioactivity in forearm microcirculation^17–18^ coupled with reduced smooth‐muscle vasodilator response to NO donors and thus have more generalized vascular dysfunction than whites.^18,30^

Our findings of reduced RHI and worse microvascular function in blacks, even after adjustment for differences in CVD risk factor burden, are consistent with those of Mulukutla et al,^31^ who also observed that black race was independently associated with lower PAT ratio across all Framingham risk strata. In contrast to that study, however, our population was younger with a lower Framingham risk score. Importantly, although baseline PVA and total hyperemic response were both lower in blacks, the peak hyperemic response was similar in blacks and whites, indicating increased resting as well as hyperemic microvascular constrictor tone in blacks. Similar reductions in resting and hyperemic blood flow have been observed secondary to aging and diabetes.^32^

Increases in CAIx and PWV have been associated with higher risk of adverse cardiovascular events and appear to be stronger predictors of outcomes than brachial pulse pressure.^33–35^ Previous studies of racial differences in large artery stiffness have been limited in sample size and by the use of alternative techniques such as carotid artery β stiffness index.^36–37^ Similarly, in prior small studies, higher AIx and PWV have been observed in blacks compared with whites.^22,38^ To the best of our knowledge, ours is the largest study to date that provides compelling evidence that black race is associated with abnormalities in indices of arterial wave reflections and stiffness, even after adjustment for traditional CVD risk factors.

The pulsatile arterial tonometer is programmed to estimate the peripheral augmentation index (PAT‐AIx) using an algorithm that transforms the digital signal. In the largest investigation to date, our study shows that PAT‐AIx is not only higher in blacks but is also associated with other conventional risk factors, as recently reported in smaller studies.^39–40^

Race‐independent associations between CVD risk factors and indices of digital and central vascular reactivity have been previously investigated. Comparable to our findings, in the largely white Framingham cohort, RHI was directly associated with age and inversely associated with total/HDL cholesterol.^8^ In addition, we calculated fRHI, an index that had a better correlation with risk factors in the Framingham study, and found similar results.^8^ In contrast to RHI, we found strong associations between other traditional CVD risk factors and indices of arterial stiffness and wave reflections. These findings illustrate that the impact of risk factors on microvascular function in the digital vascular bed differs considerably from that on central arterial stiffness, and yet both these measures are abnormal in blacks. Although other factors such as socioeconomic class and physical activity may contribute to the observed differences, adjustment for these factors did not diminish the racial differences in PAT hyperemia measured in a recent study.^31^

Understanding racial differences in endothelial function and arterial stiffness can help to identify patients at high risk for adverse outcomes including renal disease, myocardial infarction, and stroke. Vascular function measures have the advantage of integrating the time‐dependent effects of risk factors over the life span, as well as the modulatory influences of psychosocial factors and racial differences in genetic susceptibility. The striking abnormalities we observed in microvascular tone and arterial elasticity in blacks without any CVD risk factors illustrate that elements beyond conventional risk factors are important. Future studies should investigate whether incorporation of vascular function into new risk prediction models for CVD provides more accurate assessment of risk in blacks.

The strengths of our study include a large sample size of an unselected community‐based population with good representation of young, female, and black subjects. In addition, our cohort was large enough to allow the selection of a subgroup that lacked traditional risk factors for CVD, illustrating that the observed racial differences in vascular function cannot be attributed solely to differences in CVD risk factor burden. These strengths may make our findings more generalizable than previous data. In addition, ours is the first study to use multiple measurements of vascular function in the same subject, allowing for comparison of different modalities of assessing vascular function. We are also one of the first groups to report data for variables such as PAT‐AIx that have not previously been reported in large cohorts.

Limitations include the cross‐sectional design of our study, which precludes establishment of a causal relationship between risk factors and digital vascular function or arterial stiffness. Although we accounted for antihypertensive and statin medication use (<12% of our study population reported use of these medications), it is uncertain whether treatment duration and intensity influenced the vascular parameters. It is recognized that socioeconomic factors may mediate racial differences in CVD risk and that a low level of education has been associated with greater arterial stiffness.^41^ However, adding level of education in our sample did not significantly alter our findings. PWV was higher in both black men and women in the healthy subgroup, but only in men with risk factors. This may be because of technical aspects such as abdominal obesity and large bust size in women that can make distance measurements imprecise and affect PWV measurements.

In conclusion, we have demonstrated lower digital reactive hyperemia and increased arterial wave reflections and arterial stiffness in blacks compared with whites in a community‐based sample. These findings demonstrate that blacks have worse vascular function compared with whites independent of differences in CVD risk factor prevalence. Because impaired RHI and indices of arterial elasticity have been associated with worse long‐term outcomes, these measures may be useful tools for monitoring risk in blacks who have higher CVD risk compared with whites and for following response to therapy in this higher‐risk population.
